# Microporous Organic Polymers: Synthesis, Characterization, and Applications

**DOI:** 10.3390/polym11050844

**Published:** 2019-05-10

**Authors:** Johannes Carolus Jansen, Elisa Esposito, Alessio Fuoco, Mariolino Carta

**Affiliations:** 1 Institute on Membrane Technology, ITM-CNR, Via P. Bucci 17/C, 87036 Rende (CS), Italy; e.esposito@itm.cnr.it (E.E.); a.fuoco@itm.cnr.it (A.F.); 2 Department of Chemistry, College of Science, Swansea University, Grove Building, Singleton Park, Swansea SA2 8PP, UK

The presence of a certain degree of porosity in polymers is a feature that provides them with unique properties and with opportunities to be exploited in a number of technologically important applications. Depending on the size and chemical nature of the pores, they may be exploited for selective adsorption and/or storage of specific molecules; the pores may host catalytically active species or have catalytic activity themselves; they may host probes that act as sensors; pores may also provide reservoirs for controlled release of previously absorbed species; and finally, they may simply provide a selective and preferential pathway for the transport of gases, liquids, or solutes. Within this context, microporous organic polymers represent a rapidly expanding class of amorphous porous materials, composed of fully covalently bound organic building blocks. Depending on the appropriate choice of monomers, functionality, and polymerisation method, they can be prepared both as solution processable or as insoluble networked materials. Typical features of microporous organic polymers (MOPs) are pore diameters of less than 2 nm, high internal surface areas, and elevated thermal stability [1]. At the borderline between microporous and macroporous polymers we can find systems prepared by phase separation or other techniques. Typical challenges in this field are related to polymer synthesis (where the polymer type can be ladder-type polymers of intrinsic microporosity (PIMs), thermally rearrangeable (TR) polymers, or porous organic networks), to the structural characterization of the polymers and/or to the modelling of their structure and properties, and to different application types (e.g., gas sorption and storage, gas permeation, catalysis, heavy metal sorption, energy storage) and operation principles. In this special issue, we report some of the latest advances in the field of synthesis, characterisation and applications of porous or microporous organic polymers, with a total of 11 articles, two of which are reviews.

The first group represents solution-processable MOPs. These polymers may be synthesized via different routes, but the key factor is that they consist of highly contorted and rigid polymer backbones, which guarantees on the one hand an enhanced solubility in common organic solvents, and on the other hand very poor packing of the polymer in its solid state, resulting in a high free volume. This is the fundamental principle of PIMs, a novel class of polymers introduced in 2004 by Budd and co-workers [2] that, since then has become the subject of numerous studies [3]. Based on this principle, Genduso et al. report the synthesis and characterization of a novel triptycene-based solution-processable polyamide obtained via polycondensation reaction [4]. The trigonal shape of the triptycene grants an enhanced internal free volume, but the amide bond still provides a certain degree of mobility compared to other much stiffer PIMs, resulting in moderately high gas sorption capacity and gas permeability, but significantly higher selectivity than similar polyimides. Alternatively, Esposito et al. report the performance of the true polymer of intrinsic microporosity PIM-EA(H_2_)-TB, pristine and blended with Matrimid® [5]. The two polymers showed good compatibility, allowing tailoring of the properties of the blend and increasing the permeability of the pure Matrimid® by some orders of magnitude. This is a convenient alternative to the development of completely novel polymers. Dujardin et al. discuss the characteristics of a class of chemically and thermally robust norbornene based polymer membranes, crosslinked with vinylnorbornene (VNB) comonomer units [6]. Here, the permselective properties could be tailored by the VNB content in the polymer.

The second group consists of cross-linked polymers that are mostly used for gas storage or capture, rather than membrane separation, because they lack the film forming properties needed for membrane preparation. This topic is of growing importance because of the increasing pressure on the environment by human activities, which urgently necessitates the reduction of the emission of greenhouse gases, such as CO_2_, into the atmosphere and the development of alternative fuel technologies based on hydrogen or renewable energy sources [7]. Cousins and Zhang provide a review on porous organic polymers (POPs) for hydrogen storage, with theoretical considerations on storage capacity, and an extensive overview of different materials [8]. In a paper closely related to that of Dujardin et al. [6], Alentiev et al. report cross-linked polynorbornadienes for CO_2_ capture [9]. This confirms the great versatility of this very large family of polymers for different applications [10]. In another review on POPs, using covalent triazine frameworks, Zhang and Jin discuss the potential of these materials for gas adsorption, separation, and catalysis for energy and environmental applications [11]. The potential of POPs in catalysis is further discussed in detail by Wang et al. in their feasibility study on the use of these polymers as metallocene catalyst supports for ethylene polymerization [12].

Phase separation processes lead to microscopic porosity and forms the basis for the third group of materials. Under controlled conditions, block copolymers yield hierarchically porous films with nanometre-sized pores, exploited by Guo et al. for the immobilization of glucose oxidase in polystyrene-*block*-poly(4-vinyl pyridine) developed for sensor applications [13]. Somewhat analogously, Koromilas and co-workers exploit the blending of pristine and sulfonated polysulfones to control the pore structure and hydrophilicity in phase inversion membranes for water filtration [14]. Alternatively, He et al. generate porosity in microspheres by a foaming method [15]. This leads to a large central void inside the sphere and controllable microscopic voids in the sphere wall. A completely different approach to tailor porosity for controlled release systems is presented by Wakui and Aizawa [16]. Starting from a nonwoven fabric containing a drug for controlled release, CO_2_-assisted polymer compression reduces the macroscopic pores.

In conclusion, the papers in this special issue confirm that porosity in polymers, not limited to microporosity in its strictest definition, opens perspectives for numerous potential applications. They contribute to an increasingly interesting research field, which unquestionably deserves further effort. We wish to thank all the authors for contributing to this issue with their manuscripts, and the reviewers for their critical evaluations that guaranteed the quality of the individual contributions.
